# Developing emotional preparedness and mental resilience through high-fidelity simulation: a ‘bridge too far’ for institutions teaching major trauma management and mass-casualty medicine?

**DOI:** 10.1186/s12909-024-05526-8

**Published:** 2024-05-15

**Authors:** Jon Newton, Andrew D.A.C. Smith

**Affiliations:** 1 School of Health and Social Wellbeing, University of the West of England, Bristol, UK; 2 Mathematics and Statistics Research Group, University of the West of England, Bristol, UK

**Keywords:** Psychological resilience, Patient simulation, Mass-casualty incidents, Emergency medicine, Paramedics, Education

## Abstract

**Background:**

Clinical acumen represents only part of being adequately equipped to attend a major incident. The emotive sights, sounds and smells of these dynamic environments are all-encompassing experiences, and responders must also be armed with the emotional preparedness to perform their clinical or managerial duties effectively, as well as the mental resilience to facilitate professional continuance. Despite this, limited training and a sparsity of evidence exists to guide developments within this domain. Historically, major incident training has focused on clinical theory acquisition, but irrespective of how comprehensive the learning materials, they are of little consequence if tandem steps to cultivate mental resilience and emotional preparedness are absent. High-Fidelity Simulation (HFS) has a growing reputation as an effective means of bridging important gaps between theory and practice. This pilot study aimed to measure student’s self-reported perception of their readiness to respond to a major incident following a large-scale HFS.

**Methods:**

Quantitative data was obtained from a sample of 108 students undertaking paramedic science, physician associate studies and adult nursing degree programmes. A bespoke questionnaire was developed to measure self-reported clinical acumen, mental and emotional preparedness.

**Results:**

91% of students agreed the combination of theoretical training and HFS provided made them feel clinically prepared to attend a real major incident; 86% agreed this experience had developed their mental resilience and 90% agreed that they felt emotionally prepared to attend a major incident.

**Conclusion:**

Within this pilot study, the blend of theoretical training and HFS contributed to self-reported clinical acumen, mental and emotional preparation, in learners training to work in disaster environments or emergency medicine settings.

**Supplementary Information:**

The online version contains supplementary material available at 10.1186/s12909-024-05526-8.

## Background

Attending the scene of a major incident is a profound experience [1], and one that perhaps nobody can ever be truly prepared for [2]. The intensity and severity of these environments have the potential to damage the mental health of emergency service responders; irrespective of their level of experience, knowledge, or training [1, 3–6]. Major incident training is taught worldwide, yet typically represents only a small aspect of the curriculum and learning materials typically focus on its more tangible and unique facets, such as mass-casualty triage and the Joint Emergency Services Interoperability Programme (JESIP) principles [7].

Higher education delivery is more than simply introducing theoretical concepts to learners [8]; it is about facilitating opportunities for knowledge application within communities of professional practice [9, 10]. Experiential learning builds upon the pedagogy of social constructivism, behaviourism and cognitivism, with clinical placement at the core of the learning process [8, 9, 11, 12]. Safely equipping the future workforce with the requisite skillset to manage mass-casualty incidents is more challenging. Educators are well aware of the widespread benefits of embedding practice-based learning within curricula [8, 10, 13–15], although it would be neither safe or ethical to arrange ‘warzone’ placements for students, or wait for the next major incident to be declared and ask for them to be deployed.

Undertaking computer-based exams and/or completing academic assignments does not clinically and emotionally prepare learners for real-world practice within this field because it fails to cultivate the disposition of ‘antifragility’ [16]. This term depicts the notion that through exposure to stressors, volatility and randomness, growth will occur; the only caveat being that the metaphorical load placed upon a learner must not surpass their personal threshold [17]. A simple analogy of this concept is an individual lifting weights in the gym. If appropriately conducted, the process will cause microscopic tissue damage, resulting in repair and hypertrophy – and lead to developments in strength over time. In the case of training emergency service professionals, we suggest that traditional face-to-face lecturing does not adequately test clinical acumen and emotional strength without dovetailing it with hands-on practical experience, in dynamic learning environments. Technological advancements in virtual reality equipment and interactive human mannequins have acted as a catalyst in propelling innovations within simulation [18–20]. A growing body of evidence has showcased the widespread benefits of simulation learning activities within healthcare education [8, 10, 13–15, 19, 21], but studies that have explored its value within major incident training are limited to clinical acumen only and do not investigate mental resilience nor emotional preparedness [22].

The UK Department of Health, the European Union Civil Protection Knowledge Network, and the World Health Organisation recommend and utilise discussion-based learning, tabletop scenarios and live-play simulation of major incidents for training emergency responders [23–26]. There is variability in how elaborately or proficiently institutions execute mass-casualty simulation, and creating ultra-realistic, immersive exercises with a ‘movie-set’ feel is time-consuming, expensive and places a significant burden on training departments [13, 14]. Further barriers associated with health and safety, risk management, sustainability, General Data Protection Regulation (GDPR), ensuring parity and equal opportunities for every participant, further challenge implementation [5].

Training emergency service staff to be effective in a major incident is vital in today’s volatile world; the likelihood of a terrorism-related major incident is thought to be increasing in the UK and within the European Union [24, 27–30]. Emergency service personnel would be first on-scene at a major incident, some of whom will be junior or inexperienced, highlighting a need to appropriately train and equip these individuals clinically and emotionally. Responding to acts of terrorism represents just one aspect of major incident management that frontline responders could endure [23]. Accidents involving hazardous goods, road traffic collisions, aviation or rail disasters, structural collapses and natural disasters represent several further examples [31]. Major incidents typically happen quickly and unexpectedly [32] and thus, when a responder makes their way to the scene they will have had very little time to assimilate, predict and prepare for what awaits them.

Acute Stress Disorder (ASD) and Post-Traumatic-Stress-Disorder (PTSD) are known to be prevalent in emergency service workers worldwide [33–37]. Whilst these conditions are regarded as a normal reaction to an abnormal event [38, 39], PTSD predictably emerges following exposure to traumatic event/s when an individual is forced to reside in a position of “relative helplessness” [38]. Therapies to support PTSD sufferers have gained traction in recent years [40], yet primary diagnostic awareness and provision of treatment can be poor [41–43] and slow referrals or long waiting lists to see specialists can lead to mental health crises, self-harm ideation and in extreme cases result in suicide [1, 44–46]. It is apparent that a research-knowledge gap exists surrounding effective cultivation of emotional readiness in Category 1 responders (a person or professional body responsible for providing a core major incident response [47]) in readiness to attend a major incident; and limited strategies to reduce psychological harm.

Our aim was to investigate how High-Fidelity Simulation (HFS) can contribute towards developments in clinical, mental and emotional readiness to respond to a major incident, in learners training to work in emergency medicine settings. We collected data following Operation JACKSCREW, a major incident HFS, provided to healthcare students in higher education, that took place in Bristol, UK in 2022. Our objectives were (i) to pilot a questionnaire developed specifically to measure student perception of their readiness to respond to a major incident following the HFS, (ii) to report student’s self-reported readiness and experience of the HFS, and (iii) to further report self-reported clinical acumen and emotional preparedness for specific major incident protocols amongst paramedic science students.

## Methods

A six-week, fifteen credit, level six module in major incident clinical care at the University of the West of England, Bristol was composed with the intention of equipping students with the requisite level of clinical acumen, mental resilience and emotional preparedness to attend a major incident. The first author’s industry knowledge and experience within major incident management was combined with current National Ambulance Resilience Unit (NARU) protocols, the Central Government Emergency Response Training (CGERT) guideline, NHS England’s Clinical Guidelines for Major Incidents and Mass Casualty Events and the Kerslake Report to create the learning materials. The module comprises of three theoretical teaching days (plus self-study), three progressive HFS days and a 3000-word reflective assignment on experiential learning. The penultimate HFS staged was an aviation disaster, using the fuselage of a commercial airliner and 32 scripted casualties. A moulage team were brought in to simulate a range of traumatic injuries and a series of special effects were utilised to create a movie-set environment. The exercise was titled Operation JACKSCREW, and it comprised of 505 personnel and ran over a 4-day period. In addition to the aeroplane crash site, the simulation included a mock major trauma hospital, staffed with a medical team working at full capacity in a department already full of patients. Victims of the simulated aviation disaster were conveyed in blue-light ambulances. After completion of the exercise, police witness statements were collected by all in attendance. Forensic science students then investigated the simulated crash-site in which industry experts had planted evidence providing scope for these learners to uncover a detailed series of events. The final element of the simulation was a public inquiry in a courtroom setting, where core participants were called to give evidence; watched by members of the ‘deceased family’. The event has since been recognised as the UK’s largest, university-led interprofessional HFS [5].

**Fig. 1:**
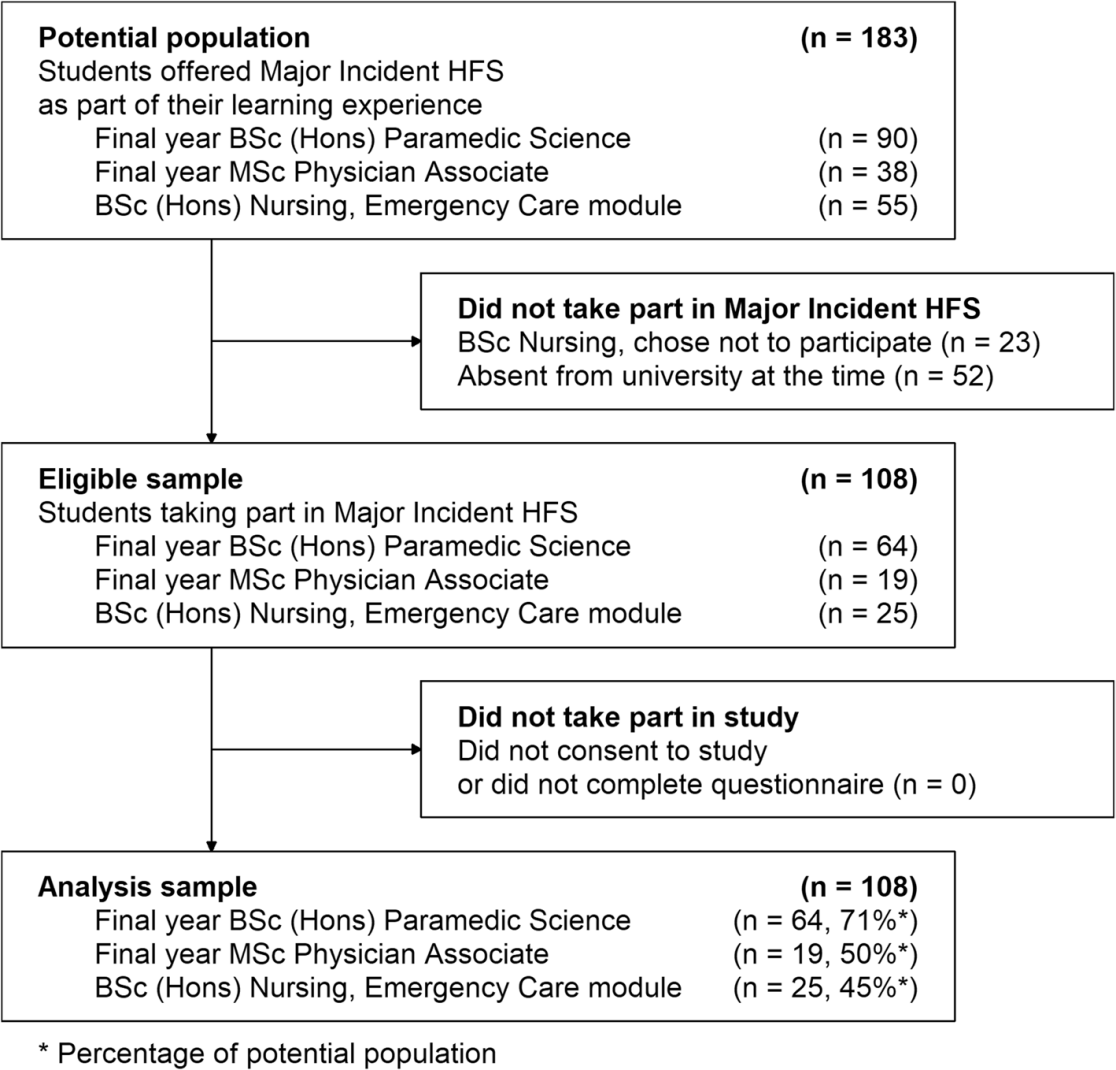
Composition of the analysis sample of 108 students who took part in the HFS

Data was collected by means of a self-completed questionnaire distributed following Operation JACKSCREW to all student participants. The potential population was all students offered the HFS as part of their learning experience, comprising all students registered on the major incident clinical care module, which was all students in two final year cohorts studying BSc (Hons) paramedic science; all students in one cohort of final year MSc physician associate students; and students in one cohort of BSc (Hons) nursing students who had selected Emergency Care as a ‘choice module’. The HFS was provided as part of the planned learning activities for students on paramedic science and physician associate programmes; students from nursing programmes were invited to take part as an optional additional learning activity. Students were not aware they would be offered the chance to participate in a research study prior to undertaking the HFS. After the HFS had concluded, all students attending were invited to take part in the research study and their informed consent was sought. Participation in this study was optional, anonymous and there was no financial or educational incentive/reward for survey completion, nor any academic/assessment consequences, to mitigate for participation bias. Those completing the survey did so as an act of goodwill; questionnaires were placed in a sealed collection box, enabling views to be expressed without the risk of feeling judged, criticised, or experiencing academic repercussions. Our sample for analysis included all students in the potential population and excluded all students who did not take part in the HFS or did not consent to the study. Figure 1 shows the composition of the potential population and the analysis sample.

To our knowledge, no previous studies have systematically addressed the psychometric properties of mental resilience or emotional preparedness using well-defined criteria against emergency service workers in disaster environments or HFS [22]. Several studies have been conducted to investigate emotional preparedness in relation to major incidents [48–50], but these are linked to the practices of social workers, which does not align with our sample population.

The initial set of items were derived by the first author, and are provided in Supplementary Material. These comprised of questions relating to clinical acumen, as well as aspects related to mental resilience and emotional preparedness. The items relating to self-reported advancements in clinical acumen focused on topics surrounding the theoretical learning provided to study participants within the major incident module (i.e. prior to undertaking Operation JACKSCREW), thus the questions were forecast against the students’ experience and level of study. The questions were comprised to ensure clinical suitability across the breadth of the different professional disciplines participating. Further depth was provided by composing supplementary questions to ask the paramedic students, specifically relating to their initial actions as first-responders. Two questions aimed to ascertain if students felt the HFS had taken them outside their comfort zone, or become overwhelmed. The rationale for measuring this is Panicucci’s zones of learning [51, 52]. The educational theory surrounding this paradigm dictates that new behaviours are elicited when working in territories which are unfamiliar, unpredictable and risky [53]. If this is achieved, learners may enter a learning (or stretch) zone – which is where personal growth occurs. If limited stimuli are provided, learners remain within their comfort zone and limited gains are achieved. In contrast, if the stimulus is too demanding the learner may bypass the learning zone and enter the panic zone (due to being overwhelmed.) This typically results in a retreat to the comfort zone. The remaining questions were designed to capture whether the HFS was perceived to develop participants mental resilience, and asked about specific situations to ascertain if they felt learning had been provided to cultivate enhancements in emotional preparedness. These questions targeted very specific areas of clinical practice surrounding decision making at a major incident scene.

Face validity of the questionnaire was revised by the second author, and 3 university colleagues who were experienced healthcare professionals/university lecturers, with insight into the module and the associated HFS. These colleagues commented on any perceived ambiguity and made suggestions to enhance the wording. The wording of each survey question was then refined to remove ambiguity and minimise potential participant confusion. Finally, non-medical personnel proof-read the questions, enabling us to further refine elements which may be perceived as unclear. One principle used to select questions and ensure face validity was that the questionnaire should aim to measure no more than three domains: clinical acumen, mental resilience, and emotional preparedness.

The questionnaire recorded baseline characteristics of the students’ gender, age, degree programme, and previous clinical experience. Subsequently, students rated their agreement on a 7-point Likert scale to statements relating to their experience of the HFS. All students were asked to rate 9 core statements plus one statement if they attended the courtroom experience, and paramedic science students were asked a further 9 statements relating to their unique experiences as first responders at a major incident. The statements are reproduced in Supplementary Material Tables 2 and 3, to show the statements and levels of agreement.

In our main analysis, we considered the sample of all students and the 9 core statements. We used principal components analysis as an exploratory factor analysis to establish how many constructs (the ‘dimensionality’) the questionnaire may measure and whether these aligned with our prior ideas. To determine whether the data fit hypothesized models of the constructs identified, we further conducted confirmatory factor analysis. To assess the reliability of the questionnaire in terms of internal consistency, we calculated Cronbach’s alpha statistic for all questions within each construct. We considered alpha above 0.5 to be ‘starting level’, above 0.7 to be acceptable and above 0.8 to be good [54, 55]. Full details of the factor and reliability analyses are provided in Supplementary Material. When reporting, we dichotomized the Likert scale score: students were deemed to have agreed with the statement if they scored in the top 3 points of the scale (i.e. ‘neither agree nor disagree’ was not counted as agreement).

We additionally reported the responses regarding the courtroom experience, and the further questions posed to paramedic science students, but did not include these in the factor or reliability analyses due to a reduced sample size. To assess whether reliability was affected by differences between the questionnaires administered (i.e. between the longer questionnaire given to paramedic science students and the shorter questionnaire given to all other participants), we performed a sensitivity analysis, repeating the reliability analysis for paramedic science students only. To assess whether differences in reliability were due to differences between the paramedic science students and other students in the sample, we compared the baseline characteristics and responses of paramedic science students with other students using chi-square tests and Welch’s test [56]. Confirmatory factor analysis took place using the R statistical package; all other analysis took place using SPSS version 29.

## Results

Although completing the questionnaire was optional, all students who took part in the HFS also consented to the study and returned the questionnaire. Our analysis sample consisted of 108 students who took part in the HFS and returned the questionnaire. Figure 1 shows how the analysis sample relates to the potential population, and Table 1 shows baseline characteristics of the analysis sample. The sample was 63% female and 73% below 26 years of age. Approximately half the sample (51%) reported no previous direct or indirect experience of a major incident in real-world practice or in a simulated environment. Table 2 shows the percentage agreement to the statements posed to all students. There were 64 students on the paramedic science programmes; Table 3 shows the percentage agreement to the statements posed to paramedic science students only.

**Table 1:** Baseline characteristics of 108 students who took part in the HFS

	*n*	(%)
Gender		
Female	68	(63.0%)
Male	40	(37.0%)
Transgender	0	(0.0%)
Not defining as either	0	(0.0%)
Prefer not to say	0	(0.0%)
Age		
Less than 26 years	79	(73.1%)
26–36 years	26	(24.1%)
More than 36 years	3	(2.8%)
Degree programme		
BSc (Hons) Paramedic Science	64	(59.3%)
BSc (Hons) Adult Nursing	25	(23.1%)
MSc Physician Associate	19	(17.6%)
Previous experiences (clinical)		
Taken part in a major incident training exercise	38	(35.2%)
Worked in A&E treating victims of a major incident	14	(13.0%)
First person on-scene at a major incident	10	(9.3%)
Dispatched to a major incident on a RRV or DCA	7	(6.5%)
Conducted a functional role at a major incident	2	(1.9%)
Major Trauma patients seen on placement		
0	31	(28.7%)
1–5	61	(56.5%)
6–10	8	(7.4%)
11 or more	8	(7.4%)
Previous experiences (reporting)		
Conducted SIEVE or SORT at a major incident	4	(3.7%)
Provided a Windscreen report to ambulance control	1	(0.9%)
Provided a METHANE report to ambulance control	0	(0.0%)
Previous experiences (medico-legal)		
Provided a Witness Statement to police	24	(22.2%)
Given evidence in court	4	(3.7%)

**Table 2:** Questionnaire responses from 108 students who took part in the HFS

Construct and statement	Responses	Agree	(%)
**Clinical acumen**			
Major trauma training left you feeling clinically prepared to support a real major incident	108	98	(90.7%)
Working alongside emergency service staff in a major incident simulation advanced understanding of joint working	105	104	(99.0%)
Major incident simulation developed critical thinking and problem-solving skills	107	100	(93.5%)
Major incident simulation developed ability to implement autonomous clinical care	107	95	(88.7%)
**Mental and emotional preparedness**			
Major incident simulation enhanced emotional preparedness and mental readiness to support a real major incident*	105	95	(90.5%)
Major incident simulation developed mental resilience	105	90	(85.7%)
Lecture material and simulation left you feeling emotionally prepared to attend a real major incident	107	96	(89.7%)
Courtroom simulation provided better emotional preparation for major incidents^†^	81	70	(86.4%)
**Discomfort**			
Felt outside comfort zone within major incident simulation	105	78	(74.2%)
Felt overwhelmed within major incident simulation	105	58	(55.2%)

Students were asked to rate the strength of their agreement using a 7-point Likert scale. Scores in the top 3 points of the scale were deemed to agree with the statement (i.e. ‘neither agree nor disagree’ was not counted as agreement)

**Table 3:** Additional questionnaire responses from 64 paramedic science students who took part in the HFS

Construct and statement	Responses	Agree	(%)
**Clinical acumen**			
Have confidence to provide a Windscreen report	61	47	(77.0%)
Major incident training improved ability to provide a METHANE report	64	58	(90.7%)
Possess clinical confidence to implement all 'Initial Actions' if first person on scene at major incident	64	42	(65.6%)
Gained clinical knowledge to conduct SIEVE	64	59	(92.2%)
Gained clinical knowledge to conduct SORT	64	56	(87.5%)
Confidence to implement JESIP	63	46	(73.0%)
Could confidently apply remaining theoretical concepts in real-world practice	64	55	(85.9%)
**Mental and emotional preparedness**			
Have emotional strength to classify adult patient as dead	64	55	(85.9%)
Have emotional strength to classify paediatric patient as dead	63	41	(65.1%)

Students were asked to rate the strength of their agreement using a 7-point Likert scale. Scores in the top 3 points of the scale were deemed to agree with the statement (i.e. ‘neither agree nor disagree’ was not counted as agreement)

Exploratory factor analysis indicated that there may be three factors measured by the questionnaire, with confirmatory factor analysis indicating a reasonable approximate fit (Supplementary Tables 1–2). The factors were: clinical acumen, relating to statements regarding clinical preparedness, understanding joint working, critical thinking and autonomous clinical care; mental and emotional preparedness; and ‘discomfort’, related to feelings of being outside of comfort zone or overwhelmed. In the reliability analysis (Supplementary Tables 3–4), statements posed to all students had a ‘starting level’ of internal consistency in the clinical acumen factor and a high acceptable level in the other factors. Statements posed only to paramedic science students had a good level of internal consistency in the clinical acumen factor.

### Clinical acumen

Over 90% of all students agreed that they felt clinically prepared to support a major incident following the training provided. Agreement with other statements of clinical acumen, that were posed to all students, ranged between 89% and 99%. For the seven statements posed only to students on paramedic science programmes, the values associated with those who agreed they felt ‘clinically prepared’, ranged from 66% to 92%. Clinical acumen was highest with regard to participants feeling accomplished in conducting a major trauma SIEVE and making a METHANE report (at least 90% agreement), and the lower percentage values attributed to conducting their initial on-scene actions and utilising JESIP (66% and 73% agreement respectively).

### Mental and emotional preparedness

Over 85% of all students agreed that the HFS developed their mental resilience, and 91% agreed it had enhanced their mental readiness to support a major incident. Approximately 90% agreed that they felt emotionally prepared to attend a major incident following the training. Out of the students that attended the courtroom simulation, 86% agreed it provided emotional preparation for major incidents. Additionally, 86% of paramedic science students agreed they had the emotional strength to classify an adult patient as dead but only 65% agreed to the equivalent statement for paediatric patients.

Discomfort: A majority (74%) of students felt outside their comfort zone within the simulation, but only 55% felt overwhelmed.

### Sensitivity analyses

The paramedic science students were more likely to be male and had slightly more previous experience than other students in the analysis sample (Supplementary Table 5). When we restricted our analysis to paramedic science students, the internal consistency was similar to our first analysis (Supplementary Table 6). Paramedic science students felt more outside their comfort zone than other students in the analysis sample, but strongly agreed that the HFS advanced their understanding of joint working. They also strongly agreed that the HFS enhanced their emotional preparedness and mental readiness (Supplementary Table 7).

## Discussion

Our pilot study indicates that the questionnaire may be able to measure self-reported development in clinical acumen, and mental and emotional preparedness, following HFS. Our principal finding was that 91% of participants agreed the combination of the theoretical training and HFS delivered made them feel clinically prepared to attend a real major incident. In addition, 86% agreed this experience had developed their mental resilience and 90% agreed that they felt emotionally prepared to attend a major incident. We suggest that HFS augments performance in learners who have received appropriate prior theoretical training whilst helping psychologically equip them to deal with the environmental stresses associated with mass-casualty scenes. These findings support that of fellow researchers working in similar fields [4, 5, 8, 10, 13–15, 18, 20]. The key strengths of this study include an multidisciplinary sample, and a highly realistic and believable scenario that subjected learners to every element of a major incident.

The strongest perceived benefit of the HFS in our study (99% agreement) was learners were given the opportunity to work alongside a wide variety of qualified emergency service personnel. Interprofessional working can be difficult to arrange due to high operational demand and limited resource availability; yet a growing body of evidence acknowledges the importance of including this aspect within healthcare simulation [5, 13, 14, 18]. Our study further re-enforces this notion. We suggest that good quality HFS includes realistic interprofessional working, satisfactorily scripted characters, and avoids breaks in role-play reality. Failings at this juncture will lead to periodic interruption whilst participants seek guidance from exercise supervisors which diminishes the value of a ‘real-time’ learning experience.

Clinical acumen amongst BSc (Hons) paramedic science students had comparatively lower results with regards to their perceived ability to implement initial on-scene actions at a major incident when in the role of first-crew on-scene (66%). This was closely followed by JESIP principles (73%). This was the most surprising finding from a teaching and learning standpoint because algorithmic working and fixed parameter actions add stability to dynamic and unpredictable environments; and in other aspects of the curriculum these tend to be favoured by paramedic trainees. The implications of these values require further assessment as these could compromise scene-safety, resource deployment, or command and control. It is unclear why learners appeared to struggle comparatively more with this element; and if this is representative of a wider problem for emergency service staff. Further evaluation beyond the parameters of this study is thus required. Finally, the lowest percentage value acquired in our study was for the trainee to have the emotional strength to classify a paediatric patient as ‘dead’ (65%). This finding is not surprising because within the Operation JACKSCREW simulation, it was noted by the lead doctor that ‘deceased children’ were being conveyed to hospital. The wider literature highlights the clinical process for declaring life extinct is robustly taught to healthcare professionals, but effectively dealing with the anxiety and grief associated with making such a decision is not [6, 57–59]. This represents a further area for research and development. Nevertheless, the ethical challenges and moral dilemmas associated with recognition of life extinct in major incidents is difficult for even experienced, highly-skilled clinicians [57–60].

We recommended that educators take steps to understand the experiences of learners undertaking HFS in order to evaluate confidence and self-assurance from both a clinical and emotional standpoint. We suggest effective learning takes place when an individual is taken outside their comfort zone, but not to a stage where the cognitive load surpasses their personal threshold. Our results suggest Operation JACKSCREW delivered this balance and as a result, anti-fragility gains were apparent. It is important to pre-empt the fact that individuals can become overwhelmed by a task or experience and if the cognitive load is too heavy this can be emotionally damaging and decrease learning. Ensuring a designated ‘Welfare Room’ is available for learners presenting as overwhelmed to ensure that dignity and 1:1 support can be provided is recommended during HFS. Participants needing this level of support are typically not (at least yet) ready for real-world practice and may require further mentoring. We found 74% of our sample felt outside their comfort zone during the HFS but only 55% reported feeling overwhelmed.

HFS is not the norm; and many institutions ensure learner competence through more traditional simulation. These exercises will typically be (a) shorter in duration [< 30 min], (b) reliant on scenario information being provided by an instructor to support activities occurring in real-time, and (c) contingent on ‘pause and reflect’ moments to capture and rectify mistakes or omissions. HFS represents the opposing end of the spectrum, by subjecting learners to scenarios which are typically: (i) comparatively longer in duration, (ii) scripted with sufficient content so that instructors do not need to supplement the scenario with information and (iii) the ‘pause and reflect’ approach is replaced with retrospective reflective learning. The greater the level of fidelity, the more likely the learner will suspend disbelief and engage; but the primary aim of HFS should be to provide learners with a truly believable and engaging lived-experience, which provides deep experiential learning opportunities.

Whilst student experience and satisfaction should remain at the epicentre of higher education delivery, educators have a responsibility to balance this with content that ensures learners are properly prepared for their chosen career. Educators need to the master the skillset required to sensitively take learners outside their comfort zone to achieve their clinical potential; and we suggest this is a prerequisite in mass-casualty HFS delivery. It is important for educators within this field to appreciate they are training students to become real-world responders. Burnout and attrition amongst healthcare providers is a global issue [61, 62]; and experience of attending critical or major incidents a noteworthy contributor [63]. Building resilience within the current and future workforce should therefore be a strategic priority. Facilitating ‘easy exits’ for students struggling to cope within the parameters of a well-run simulation is ultimately counterproductive, because it inhibits the development of both emotional intelligence and critical thinking skills.

Despite the encouraging results our study has generated, HFS should not be regarded ‘gold-standard’. Successful simulation is dependent on first instilling the requisite level of theoretical knowledge and competence, in order to maximise effectiveness. These key findings have widespread implications for educators and policymakers alike. It is also important to appreciate that even when knowledge and competence are present, some will struggle to cope with the inherent pressures of HFS and support will be needed. Reinforcing the notion that simulation provides a ‘safe space’ to learn and make mistakes is essential; and educators must reassure learners that making mistakes within these controlled environments is part of the learning process and devoid of the adverse consequences associated with that of real-world practice error or omission.

## Limitations

Our sample size of 108 participants could be considered poor, although there is no consensus on the minimum sample size required to validate a questionnaire [54]. Our respondent-to-item ratio of approximately 10:1 in the first analysis would be considered adequate against some benchmarks [54]. Our sample size of 64 in the second analysis is smaller still. Largely owing to COVID-19, some students did not attend every lecture or HFS provided in the module and thus, parity in learning opportunity was not uniform across the study sample. This was mitigated to some extent by providing e-learning resources to all participants. Over 30 students who were eligible to participate did not attend university at the time (due to a range of personal circumstances) and were therefore unable to participate in the study. This therefore resulted in a smaller sample size than originally intended. A small amount of data was also missing (7.4%) which relates to a series of study questions that were not answered by participants. It would appear this happened when a participant did not realise their questionnaire was printed ‘double-sided’ and therefore these individuals inadvertently skipped a page. Despite this, the missing data is most likely absent completely at random and missingness will have negligible impact on the results. In addition, although participation in the HFS varied between degree programmes (71.1% in paramedic science and 47.3% in other programmes), we observed few differences between the programmes in our sensitivity analysis, mitigating concerns about bias due to differing levels of representation in our sample. We were fortunate that all students taking part in the HFS chose to consent to the study, mitigating the risk of selection bias. The study questionnaire was answered by eligible participants at a similar point in time, and whilst each learner selected their answers autonomously, there was scope for these to be peer-guided or influenced by colleagues around them.

A further limitation is that our sample began their undergraduate or postgraduate studies during the COVID-19 pandemic and thus these learners have been subjected to a period of significantly disrupted teaching and comparatively sub-optimal clinical placement opportunities. As a result, the amount of patient contact time they had experienced prior to undergoing our major incident training had been limited due to some ambulance and hospital placements being cancelled. Finally, feelings and viewpoints captured on questionnaires are representative of a specific moment in time; and human emotions are malleable and may change over time. It is also noteworthy that the many interpersonal interactions between student participants, simulated patients and qualified staff had scope to positively or negatively influence experiences and perceptions.

Our questionnaire was developed in-house specifically for this study. Resources were not available to convene an expert panel to review the face validity of the questions, as is often recommended [54], nor to conduct a structured iterative process of questionnaire development with experts, which could improve content and criterion validity [64]. It must be noted that, in our sample, the questionnaire had only minimally adequate reliability in the measure of clinical acumen. The reliability improved in the questionnaire given to paramedic science students. Our sensitivity analysis showed it was the additional questions given to paramedic science students, rather than any differences between paramedic science students and other students, that caused this improvement. Our questionnaire measures self-reported perceptions about clinical acumen, and mental and emotional preparedness. Although our questionnaires were completed anonymously, social desirability bias may cause participants to rate their experiences more positively than their true experience. We were not able to assess the criterion validity of our questionnaire i.e. to assess the correlation with an external outcome. A possible external outcome that measures clinical acumen would be the students’ assessment grades, but these could not be obtained while simultaneously assuring students of the anonymity of their questionnaire responses. A possible external outcome that measures mental resilience and emotional preparedness might be future staff retention within the emergency services. There are some aspects of mental and emotional preparedness that our questionnaire did not measure, for example we did not measure a learner’s ability to ‘bounce back’ following adversity and the relationship this has with professional continuance – a follow-up study would be necessary to measure this.

## Conclusion

Within this pilot study, the theoretical training and HFS provided were observed to contribute towards self-perceived developments in clinical acumen, and mental and emotional preparedness, amongst learners training to work in emergency medicine settings. Our sample size is insufficient to propose our results are generalisable; nevertheless, the skills and knowledge acquired by this group of learners occasioned from a positive learning experience, sufficient enough to influence future practice. We further conclude that self-perceived levels of clinical acumen and emotional preparedness among student paramedics (using specific major incident protocols) were also developed.

The highest self-reported area of learner development was identified in the response to the question that, working alongside emergency service staff in major incident simulation advanced understanding of joint working. The lowest self-reported area of learner development was possessing sufficient emotional strength to classify a paediatric patient as deceased, closely followed by feeling clinical confidence to implement the first person-on-scene ‘initial actions’ at a major incident. These two elements of the curriculum represent key areas for further evaluation and development.

To enhance future teaching and learning practices we suggest that when engaging scenarios are coupled with heightened levels of environmental realism, believability is elevated and deeper experiential learning is facilitated. Orchestrating HFS which allow learners to manage events in real-time, invokes emotional responses which embeds knowledge retention, retrieval and performance. Despite this, skills gained by learners from HFS may not necessarily translate to real-world environments.

In summary, a well-considered blend of theoretical teaching and HFS has scope to improve clinical acumen, and mental and emotional preparedness, in both undergraduate and postgraduate learners training to work in disaster environments or emergency medicine settings. Further research is now required to strengthen the breadth of literature available within this specialist field. Our pioneering approach to teaching and learning may also possess scope to mitigate for the occurrence of psychological trauma in Category 1 Responders.

### Supplementary Information

**Supplementary Material 1. **

## Data Availability

The data that support the findings of this study are available from the corresponding author upon reasonable request. All data associated with this manuscript has been stored in a repository at the University of the West of England.

## Abbreviations

HFS: High-Fidelity Simulation
JESIP: Joint Emergency Services Interoperability Programme
GDPR: General Data Protection Regulation
ASD: Acute Stress Disorder
PTSD: Post-traumatic Stress Disorder
NARU: National Ambulance Resilience Unit
CGERT: Central Government Emergency Response Training
