# Supplementary material for: Developing emotional preparedness and mental resilience through high-fidelity simulation: a ‘bridge too far’ for institutions teaching major trauma management and mass-casualty medicine?
Source: BMC Med Educ. 2024 May 15;24:544. doi: 10.1186/s12909-024-05526-8 (PMC11097583; doi:10.1186/s12909-024-05526-8)
Supplement: Supplementary file 1 — Supplementary Material 1. [file 12909_2024_5526_MOESM1_ESM.docx]

**Initial pool of questionnaire items**

The initial questions written by the first author, before they were assessed for face validity, ambiguity and wording, are reproduced below. For ease of reference, the items are annotated with the question number from the final questionnaire (see next section), if that item was included in the final questionnaire in some form.

1. To what extent do you agree or disagree that you have the confidence to provide a Windscreen Report when arriving at critical incidents?

2. To what extent do you agree or disagree that the knowledge you have gained from your major incident training has improved your ability to know how/when to provide a METHANE report?

3. To what extent do you agree or disagree that if you were ‘first-person-on-scene’ at a future major incident you possess the clinical confidence to implement all of the ‘Initial Actions’ – and refrain from treating casualties until back-up arrives?

4. To what extent do you agree or disagree that you have gained the clinical knowledge to effectively conduct a Triage SIEVE at mass-casualty incidents?

5. To what extent do you agree or disagree that you have gained the clinical knowledge to effectively conduct a Triage SORT at mass-casualty incidents?

6. To what extent do you agree or disagree that working alongside fellow emergency service providers and staff from other healthcare disciplines within the major incident simulation/s has led to advancements in your understanding of joint working strategies?

7. To what extent do you agree or disagree that you have gained sufficient confidence to implement JESIP in real-world practice?

8. To what extent do you agree or disagree that you could confidently apply the remaining theoretical concepts and other knowledge gained from this module in real-world practice?

9. To what extent do you agree or disagree that the combination of lectures and simulations included within this module have left you feeling “as clinically prepared as possible” to attend a real major incident?

10. To what extent do you agree or disagree that the sights, sounds, smells and/or the dynamic environment created within the major incident simulations took you outside your comfort zone?

(12) To what extent do you agree or disagree that on reflection, being taken outside your comfort zone within the major incident simulations has led to developments in your mental resilience? If you did not feel outside your comfort zone in any of the major incident simulations then please write N/A next to this question.

11. To what extent do you agree or disagree that the sights, sounds, smells and/or the dynamic environment created within the major incident simulations made you feel overwhelmed on one or more occasions?

(12) To what extent do you agree or disagree that on reflection, feeling overwhelmed within the major incident simulations has led to developments in your mental resilience? If you did not feel overwhelmed in any of the major incident simulations then please write N/A next to this question.

12. To what extent do you agree or disagree that the major incident simulations have developed your mental resilience?”.

13. To what extent do you agree or disagree that this module’s face-to-face lectures and supporting learning materials have enhanced your existing level of emotional preparedness and mental readiness to attend a real major incident?

14. To what extent do you agree or disagree that you have the emotional strength to classify an adult patient as ‘DEAD’ using a Triage Card at a major incident and move on to assess the next patient (despite the associated moral/ethical challenges)?

15. To what extent do you agree or disagree that you have the emotional strength to classify a paediatric patient as ‘DEAD’ using a Triage Card at a major incident and move on to assess the next patient (despite the associated moral/ethical challenges)?

16. To what extent do you agree or disagree that the major incident simulations you participated in have further developed your ability to implement critical thinking and problem-solving skills in high-pressure situations?

17. To what extent do you agree or disagree that the major incident simulations you participated in have further developed your ability to implement autonomous clinical care in high-pressure situations?

18. To what extent do you agree or disagree that the courtroom simulation provided a valuable opportunity to better emotionally prepare yourself for the medico-legal aspects of major incidents? If you did not attend the courtroom simulation, please write N/A next to this question.

19. To what extent do you agree or disagree that the combination of lectures and simulations included in the major incident module have left you feeling “as emotionally prepared as possible” to attend a real major incident?

**Final questionnaire items**

The final questionnaire included questions collecting baseline characteristics (Table 1), and the following questions that were all rated on the same 7-point Likert scale. The 19 questions below were posed (with this numbering) to paramedic science students. The questions marked with asterisks (*) were posed to all students with appropriate changes to wording where necessary.

1. To what extent do you agree or disagree that you have the confidence to provide a Windscreen Report when arriving at critical incidents?

2. To what extent do you agree or disagree that the knowledge you have gained from your major incident training has improved your ability to know how/when to provide a METHANE report?

3. To what extent do you agree or disagree that if you were ‘first-person-on-scene’ at a future major incident you possess the clinical confidence to implement all of the ‘Initial Actions’ – and refrain from treating casualties until back-up arrives?

4. To what extent do you agree or disagree that you have gained the clinical knowledge to effectively conduct a Triage SIEVE at mass-casualty incidents?

5. To what extent do you agree or disagree that you have gained the clinical knowledge to effectively conduct a Triage SORT at mass-casualty incidents?

6*. To what extent do you agree or disagree that working alongside fellow emergency service providers and staff from other healthcare disciplines within the major incident simulation/s has led to advancements in your understanding of joint working strategies?

7. To what extent do you agree or disagree that you have gained sufficient confidence to implement JESIP in real-world practice?

8. To what extent do you agree or disagree that you could confidently apply the remaining theoretical concepts and other knowledge gained from this module in real-world practice?

9*. To what extent do you agree or disagree that the combination of lectures and simulations included within this module have left you feeling “as clinically prepared as possible” to attend a real major incident? *

10*. To what extent do you agree or disagree that you felt outside your comfort zone within the module’s major incident simulations?

11*. To what extent do you agree or disagree that you felt overwhelmed within the module’s major incident simulations?

12*. To what extent do you agree or disagree that the module’s major incident simulations have developed your mental resilience?

13*. To what extent do you agree or disagree that this module’s face-to-face lectures and supporting learning materials have enhanced your existing level of emotional preparedness and mental readiness to attend a real major incident?

14. To what extent do you agree or disagree that you have the emotional strength to classify an adult patient as ‘DEAD’ using a Triage Card at a major incident and move on to assess the next patient (despite the associated moral/ethical challenges)?

15. To what extent do you agree or disagree that you have the emotional strength to classify a paediatric patient as ‘DEAD’ using a Triage Card at a major incident and move on to assess the next patient (despite the associated moral/ethical challenges)?

16*. To what extent do you agree or disagree that the major incident simulations you participated in have further developed your ability to implement critical thinking and problem-solving skills in high-pressure situations?

17*. To what extent do you agree or disagree that the major incident simulations you participated in have further developed your ability to implement autonomous clinical care in high-pressure situations?

18*. To what extent do you agree or disagree that the courtroom simulation provided a valuable opportunity to better emotionally prepare yourself for the medico-legal aspects of major incidents? If you did not attend the courtroom simulation, please write N/A next to this question.

19*. To what extent do you agree or disagree that the combination of lectures and simulations included in the major incident module have left you feeling “as emotionally prepared as possible” to attend a real major incident (i.e. in the role of a newly qualified paramedic)?

1 = Strongly Disagree, 2 = Disagree, 3 = Disagree to some extent, 4 = Undecided, 5 = Agree to some extent, 6 = Agree, 7 = Strongly Agree

To assist with our discussions on internal validity, we hypothesized that this set of items would not measure more than three constructs. We hypothesized that questions 1-9, 16-17 would measure clinical acumen, questions 9-13 would measure mental resilience, and questions 13-15, 18-19 would measure emotional preparedness.

**Exploratory factor analysis**

To establish the dimensionality of the constructs being measured by the questionnaire, we conducted principal components analysis (PCA). It is necessary to conduct PCA on a complete cases basis: if we were to include questionnaire statements that were not completed by all participants this would reduce the sample size while removing data from completed statements. In order to maintain a reasonable sample size we included 9 of the core statements in the PCA, removing the statement pertaining to the courtroom scenario (which was not attended by all students) and the statements posed only to participants on the paramedic science programme. This resulted in a sample size of 104 students for the PCA.

As all responses were measured on the same 7-point Likert scale, we chose not to rescale them and therefore conducted PCA on the covariances, rather than correlations, of the responses. We chose the number of components to present based on retaining all components with an eigenvalue greater than 1. We performed varimax rotation to make it clearer which statements loaded onto which factors. We calculated factor loadings (the correlation between each response and the extracted components) and considered a loading of greater than 0.4 in magnitude to indicate that a response loaded onto a particular component [1].

The PCA identified 3 components that accounted for 73% of the variance of the 9 statements. The factor loadings for the rotated solution are shown in Supplementary Table 1. The first factor, explaining 38% of the variance, loaded only on the two statements regarding feeling outside of their comfort zone or overwhelmed during the HFS. These statements did not load onto either of the other two factors. The second factor, explaining 23% of the variance, loaded on the statements regarding critical thinking, autonomous clinical care, mental resilience and emotional preparedness. The third factor, explaining 12% of the variance, also loaded on the statements regarding critical thinking and autonomous clinical care as well as clinical preparedness.

**Confirmatory factor analysis**

Having identified up to three constructs that the questionnaire may be measuring, we conducted confirmatory factor analysis (CFA) to determine whether the data fit a hypothesized model. We tested a three-factor model suggested by the PCA, with one modification. The items relating to critical thinking and autonomous clinical care loaded onto two factors. In our CFA three-factor model, we grouped these items together with clinical preparedness and joint working, guided by our a priori understanding of clinical acumen being separate from mental resilience and emotional preparedness.

We identified our three factors as ‘clinical acumen’, ‘mental and emotional preparedness’ and ‘discomfort’. The questionnaire statements, and the hypothesized factor they relate to, can be seen in Supplementary Table 2.

We conducted CFA using the lavaan package version 0.6-16 in R version 4.2.0. Our initial CFA failed to numerically converge due to having one factor with only two items (outside comfort zone and overwhelmed), which causes the model to not be identified. We therefore had to conduct CFA on this factor separately.

Supplementary Table 2 shows the model fits and coefficients. The model indicated an adequate fit between hypothesized and fitted model (χ(13) = 18.797, p = 0.130) and the root mean square error of approximation (0.065) was inside the range for a reasonable approximate fit [2].

**Reliability analysis**

To assess the reliability of the questionnaire in terms of internal consistency, we calculated Cronbach’s alpha statistic for all questions within each of the three factors identified in the confirmatory factor analysis. To assess whether inclusion of a particular statement harmed reliability, we further calculated Cronbach’s alpha if each statement were removed in turn. We considered alpha above 0.5 to be ‘starting level’, above 0.7 to be acceptable and above 0.8 to be good [3,4]. To assess whether reliability was affected by differences between the questionnaires administered (i.e. between the longer questionnaire given to paramedic science students and the shorter questionnaire given to all other participants), we performed a sensitivity analysis, repeating the reliability analysis for paramedic science students only. To assess whether differences in reliability were due to differences between the paramedic science students and other students in the sample, we compared the baseline characteristics and responses of paramedic science students with other students using chi-square tests and Welch’s test [5].

Supplementary Table 3 shows Cronbach’s alpha for all students and the 9 core statements. The clinical acumen factor statements had a ‘starting level’ of internal consistency, the mental and emotional preparedness statements had an acceptable level of internal consistency, and the discomfort statements had approaching a good level of internal consistency. There was no single statement which, when removed, caused an increase in internal consistency.

To assess the reliability of the additional questions (the statement pertaining to the courtroom scenario and the statements posed only to participants on the paramedic science programme) it was necessary to nominate a factor for each question to belong to. We used our a priori understanding to place the questions relating to unique experiences as first responders at a major incident into the clinical acumen factor, and the questions that specifically mentioned ‘emotional preparation’ or ‘emotional strength’ into the mental and emotional preparedness factor.

Supplementary Table 4 shows Cronbach’s alpha for the additional questions. The clinical acumen factor statements had a good level of internal consistency. The mental and emotional preparedness statements had a high starting level of internal consistency; removing the statement regarding the courtroom simulation resulted in a good level of internal consistency.

**References**

[1] Stevens JP (1992) Applied multivariate statistics for the social sciences (2nd edition). Hillsdale, NJ:Erlbaum

[2] Kline, R. B. (2016). Principles and Practice of Structural Equation Modeling (4th ed.)

[3] Tsang, S. *et al*. (2017) “Guidelines for developing, translating, and validating a questionnaire in perioperative and pain medicine,” *Saudi Journal of Anaesthesia*, 11(Suppl 1), S80-89. Available at https://doi.org/10.4103%2Fsja.SJA_203_17

[4] Streiner, D. (2003) “Starting at the beginning: An introduction to coefficient alpha and internal consistency” *Journal of Personality Assessment*, 80(1), pp. 99–103. Available at https://doi.org/10.1207/S15327752JPA8001_18

[5] Derrick, B. and White, P. (2017) Comparing two samples from an individual Likert question. International Journal of Mathematics and Statistics 18 (3), pp. 1-13.

Supplementary Table 1: Exploratory factor analysis (principal components analysis) of 104 complete responses to a questionnaire completed by students who took part in the high-fidelity mass-casualty simulation and responded to the subsequent questionnaire.

|  | Factor loadings | | |
| --- | --- | --- | --- |
|  | Factor 1 | Factor 2 | Factor 3 |
| Variation explained | 38% | 23% | 12% |
| Statement |  |  |  |
| Major trauma training left you feeling clinically prepared to support a real major incident | -0.030 | 0.064 | 0.764 |
| Working alongside emergency service staff in MIS advanced understanding of joint working | 0.003 | 0.299 | 0.022 |
| Major incident simulation developed critical thinking and problem-solving skills | 0.090 | 0.591 | 0.403 |
| Major incident simulation developed ability to implement autonomous clinical care | 0.000 | 0.513 | 0.502 |
| Major incident simulation developed mental resilience | 0.186 | 0.857 | 0.030 |
| MIS enhanced emotional preparedness and mental readiness to support a real major incident | -0.119 | 0.872 | 0.083 |
| Felt outside comfort zone within major incident simulation | 0.915 | -0.031 | 0.225 |
| Felt overwhelmed within major incident simulation | 0.911 | 0.119 | -0.248 |
| Lecture material and simulation left you feeling emotionally prepared to attend a real major incident | 0.061 | 0.609 | 0.386 |

Supplementary Table 2: Confirmatory factor analysis of 104 complete responses to a questionnaire completed by students who took part in the high-fidelity mass-casualty simulation and responded to the subsequent questionnaire

|  | Factor coefficients | | |
| --- | --- | --- | --- |
|  | Clinical acumen | Mental and emotional preparedness | Discomfort |
| Statement |  |  |  |
| Major trauma training left you feeling clinically prepared to support a real major incident | 1.000 |  |  |
| Working alongside emergency service staff in MIS advanced understanding of joint working | 0.705 p = 0.046 |  |  |
| Major incident simulation developed critical thinking and problem-solving skills | 2.277 p = 0.007 |  |  |
| Major incident simulation developed ability to implement autonomous clinical care | 2.160 p = 0.008 |  |  |
| MIS enhanced emotional preparedness and mental readiness to support a real major incident |  | 1.000 |  |
| Major incident simulation developed mental resilience |  | 0.933 p < 0.001 |  |
| Lecture material and simulation left you feeling emotionally prepared to attend a real major incident |  | 0.686 p < 0.001 |  |
| Felt outside comfort zone within major incident simulation |  |  | 1.000 |
| Felt overwhelmed within major incident simulation |  |  | 1.065  * |
| Model fit* | χ(13) = 18.797, p = 0.130 | | |
| Root mean square error of approximation (RMSEA)* | 0.065 |  |  |

* Model did not converge when Discomfort factor was included, due to this factor only having two items. The Model fit and RMSEA are obtained from a model excluding this factor, and statistical inference is not possible for items in this factor.

Supplementary Table 3: Reliability metrics in questionnaire responses from 108 students who took part in the high-fidelity mass-casualty simulation.

| Factor and statement | Cronbach’s alpha for whole factor (complete cases) | Cronbach’s alpha if statement removed |
| --- | --- | --- |
| **Clinical acumen** | 0.571 (n = 104) |  |
| Major trauma training left you feeling clinically prepared to support a real major incident |  | 0.606 |
| Working alongside emergency service staff in MIS advanced understanding of joint working |  | 0.595 |
| Major incident simulation developed critical thinking and problem-solving skills |  | 0.375 |
| Major incident simulation developed ability to implement autonomous clinical care |  | 0.364 |
| **Mental and emotional preparedness** | 0.779 (n = 104) |  |
| MIS enhanced emotional preparedness and mental readiness to support a real major incident |  | 0.599 |
| Major incident simulation developed mental resilience |  | 0.701 |
| Lecture material and simulation left you feeling emotionally prepared to attend a real major incident |  | 0.779 |
| **Discomfort** | 0.790 (n = 105) |  |
| Felt outside comfort zone within major incident simulation |  | N/A |
| Felt overwhelmed within major incident simulation |  | N/A |

Supplementary Table 4: Reliability metrics in additional questionnaire responses from 64 paramedic science students who took part in the high-fidelity mass-casualty simulation.

| Factor and statement | Cronbach’s alpha for whole construct (complete cases) | Cronbach’s alpha if statement removed |
| --- | --- | --- |
| **Clinical acumen** | 0.881 (n = 60) |  |
| Have confidence to provide a Windscreen report |  | 0.861 |
| Major incident training improved ability to provide a METHANE report |  | 0.852 |
| Possess clinical confidence to implement all 'Initial Actions' if first person on scene at major incident |  | 0.858 |
| Gained clinical knowledge to conduct SIEVE |  | 0.865 |
| Gained clinical knowledge to conduct SORT |  | 0.893 |
| Confidence to implement JESIP |  | 0.860 |
| Could confidently apply remaining theoretical concepts in real-world practice |  | 0.857 |
| **Mental and emotional preparedness** | 0.635 (n = 61) |  |
| Courtroom simulation provided better emotional preparation for major incidents |  | 0.868 |
| Have emotional strength to classify adult patient as dead |  | 0.317 |
| Have emotional strength to classify paediatric patient as dead |  | 0.234 |

Supplementary Table 5. Comparison of baseline characteristics between paramedic science students and other students, in 108 students who took part in the high-fidelity mass-casualty simulation.

|  | Paramedic science | | Other students | | Comparison |
| --- | --- | --- | --- | --- | --- |
|  | *n* | (%) | *n* | (%) |  |
| Programme  Paramedic science  Other | 64 | (59.3%) | 44 | (40.7%) |  |
| Gender  Female  Male | 35  29 | (54.7%)  (45.3%) | 33  11 | (75.0%)  (25.0%) | χ^2­^(1) = 4.613 (p = 0.032) |
| Age  Less than 26 years  More than 26 years | 49  15 | (76.6%)  (23.4%) | 30  14 | (68.2%)  (31.8%) | χ^2­^(1) = 0.932 (p = 0.334) |
| Previous experiences (clinical)  Taken part in major incident training exercise  Worked in A&E treating victims of major incident  First person on-scene at major incident  Dispatched to major incident on RRV or DCA  Conducted a functional role at a major incident | 32  3  5  7  2 | (50.0%)  (4.7%)  (7.8%)  (10.9%) (3.1%) | 6  11  5  0  0 | (13.6%)  (25.0%)  (11.4%)  (0.0%)  (0.0%) |  |
| Major Trauma patients seen on placement  0  1-5  6 or more | 15  45  4 | (23.4%)  (70.3%)  (6.3%) | 16  16  12 | (36.4%)  (36.4%)  (27.3%) |  |
| Previous experiences (reporting)  Conducted SIEVE or SORT at a major incident  Provided a Windscreen report to ambulance control  Provided a METHANE report to ambulance control | 3  1  0 | (4.7%)  (1.6%)  (0.0%) | 1  0  0 | (2.3%)  (0.0%)  (0.0%) |  |
| Previous experiences (medico-legal)  Provided a Witness Statement to police  Given evidence in court | 20  3 | (31.3%)  (4.7%) | 4  1 | (9.1%)  (2.3%) |  |
| Derived: Previous experience of a major incident  Previous experience in real life or simulation  No previous experience | 35  29 | (54.7%)  (45.3%) | 18  26 | (40.9%)  (59.1%) | χ^2­^(1) = 1.981 (p = 0.159) |

Comparisons are made using the chi-square test.

Supplementary Table 6: Reliability metrics in questionnaire responses from 64 paramedic science students who took part in the high-fidelity mass-casualty simulation, examining only the statements posed to all students.

| Factor and statement | Cronbach’s alpha for whole factor (complete cases) | Cronbach’s alpha if statement removed |
| --- | --- | --- |
| **Clinical acumen** | 0.559 (n = 64) |  |
| Major trauma training left you feeling clinically prepared to support a real major incident |  | 0.578 |
| Working alongside emergency service staff in MIS advanced understanding of joint working |  | 0.581 |
| Major incident simulation developed critical thinking and problem-solving skills |  | 0.389 |
| Major incident simulation developed ability to implement autonomous clinical care |  | 0.354 |
| **Mental and emotional preparedness** | 0.818 (n = 64) |  |
| MIS enhanced emotional preparedness and mental readiness to support a real major incident |  | 0.652 |
| Major incident simulation developed mental resilience |  | 0.790 |
| Lecture material and simulation left you feeling emotionally prepared to attend a real major incident |  | 0.790 |
| **Discomfort** | 0.738 (n = 64) |  |
| Felt outside comfort zone within major incident simulation |  | N/A |
| Felt overwhelmed within major incident simulation |  | N/A |

Students were asked to rate the strength of their agreement using a 7-point Likert scale.

Supplementary Table 7: Comparison of questionnaire responses between paramedic science students and other students, from 108 students who took part in the high-fidelity mass-casualty simulation.

|  | Paramedic science | | | Other students | | | Comparison |
| --- | --- | --- | --- | --- | --- | --- | --- |
| Construct and statement | Responses | Agree | (%) | Responses | Agree | (%) |  |
| **Clinical acumen** |  |  |  |  |  |  |  |
| Major trauma training left you feeling clinically prepared to support a real major incident | 64 | 62 | (96.9%) | 44 | 36 | (81.8%) | t = 1.746 (p = 0.085) |
| Working alongside emergency service staff in MIS advanced understanding of joint working | 64 | 63 | (98.4%) | 41 | 41 | (100%) | t = -4.052 (p < 0.001) |
| Major incident simulation developed critical thinking and problem-solving skills | 64 | 61 | (95.3%) | 43 | 39 | (90.7%) | t = -0.840 (p = 0.404) |
| Major incident simulation developed ability to implement autonomous clinical care | 64 | 60 | (93.8%) | 43 | 35 | (81.4%) | t = 1.298 (p = 0.199) |
| **Mental and emotional preparedness** |  |  |  |  |  |  |  |
| MIS enhanced emotional preparedness and mental readiness to support a real major incident | 64 | 56 | (87.5%) | 41 | 39 | (95.1%) | t = -2.547 (p = 0.012) |
| Major incident simulation developed mental resilience | 64 | 53 | (82.8%) | 41 | 37 | (90.2%) | t = -1.781 (p = 0.078) |
| Lecture material and simulation left you feeling emotionally prepared to attend a real major incident | 64 | 58 | (90.6%) | 43 | 38 | (88.4%) | t = 0.777 (p = 0.439) |
| Courtroom simulation provided better emotional preparation for major incidents | 62 | 52 | (83.9%) | 19 | 18 | (94.7%) | t = -0.215 (p = 0.831) |
| **Discomfort** |  |  |  |  |  |  |  |
| Felt outside comfort zone within major incident simulation | 64 | 54 | (84.4%) | 41 | 24 | (58.5%) | t = 2.931 (p = 0.005) |
| Felt overwhelmed within major incident simulation | 64 | 31 | (48.4%) | 41 | 17 | (41.5%) | t = 1.428 (p = 0.157) |

Students were asked to rate the strength of their agreement using a 7-point Likert scale. Scores in the top 3 points of the scale were deemed to agree with the statement (i.e. ‘neither agree nor disagree’ was not counted as agreement).
Comparisons are made using Welch’s t test.
